# Tall fescue seed extraction and partial purification of ergot alkaloids

**DOI:** 10.3389/fchem.2014.00110

**Published:** 2014-12-11

**Authors:** Huihua Ji, F. Fannin, J. Klotz, Lowell Bush

**Affiliations:** ^1^Kentucky Tobacco Research and Development Center, University of KentuckyLexington, KY, USA; ^2^Department of Plant and Soil Sciences, University of KentuckyLexington, KY, USA; ^3^Forage Animal Production Research Unit, Agricultural Research Services, United States Department of AgricultureLexington, Kentucky, USA

**Keywords:** ergovaline, ergovalinine, alkaloid extraction, ergopeptine alkaloids, epimerization

## Abstract

Many substances in the tall fescue/endophyte association (*Schedonorus arundinaceus*/*Epichloë coenophiala*) have biological activity. Of these compounds only the ergot alkaloids are known to have significant mammalian toxicity and the predominant ergot alkaloids are ergovaline and ergovalinine. Because synthetically produced ergovaline is difficult to obtain, we developed a seed extraction and partial purification protocol for ergovaline/ergovalinine that provided a biologically active product. Tall fescue seed was ground and packed into several different sized columns for liquid extraction. Smaller particle size and increased extraction time increased efficiency of extraction. Our largest column was a 114 × 52 × 61 cm (W × L × D) stainless steel tub. Approximately 150 kg of seed could be extracted in this tub. The extraction was done with 80% ethanol. When the solvent front migrated to bottom of the column, flow was stopped and seed was allowed to steep for at least 48 h. Light was excluded from the solvent from the beginning of this step to the end of the purification process. Following elution, ethanol was removed from the eluate by evaporation at room temperature and the resulting syrup was freeze-dried. About 80% recovery of alkaloids was achieved with 18-fold increase in concentration of ergovaline. Initial purification of the dried product was accomplished by extracting with hexane/water (6:1, v/v). The aqueous fraction was extracted with chloroform, the aqueous layer discarded, after which the chloroform was removed with a resulting 20-fold increase of ergovaline. About 65% of the ergovaline was recovered from the chloroform residue for an overall recovery of 50%. The resultant partially purified ergovaline had biological activities in *in vivo* and *in vitro* bovine bioassays that approximate that of synthetic ergovaline.

## Introduction

There have been many name changes for both tall fescue and the endophyte in recent years, but *Schedonorus arundinaceus and Epichloë coenophiala* are the most generally accepted at present. The biologically active substances include the pyrrolizidine and ergot alkaloids plus peramine. Of these compounds only the ergot alkaloids have significant mammalian toxicity and the predominant, 84–97%, of the ergot alkaloids are ergovaline and ergovalinine (Lyons et al., 1986). These two ergopeptine alkaloids are isomers and in equilibrium depending upon the environment in which they are contained (Smith and Shappell, 2002). The cyclo-tripeptide of these two alkaloids is the amino group of alanine attached to the ergolene ring plus valine and proline. Concentration of alkaloid accumulation in the plant is dependent upon the growing conditions for the association. Generally, higher nitrogen fertilization, clipping frequency and cooler temperatures will increase accumulation of ergovaline in vegetative tissues (Bush and Fannin, 2009). Ergovaline has been shown to be a vasoconstrictor of the bovine lateral saphenous vein (Klotz et al., 2007) and much more potent than other alkaloids present in the grass/endophyte association with constriction induced at 10^−7^ M (see Strickland et al., 2009 for more details). A significant observation is that low amounts of ergovaline are required to elicit a response in many bioassays but sufficient pure ergovaline has not been available to conduct *in vivo* assays. Ergovaline is the more biologically active of the two isomers and neither are easily chemically synthesized. Chemical standards for analytical assays or for bioassays are very difficult to obtain. Ergovaline may be quantified in extracts from endophyte infected grasses by HPLC with fluorescent detection or by HPLC with a mass spectrometer detector. Both of these provide high sensitivity and selectivity in the determination. For analytical purposes solvent extractions from grass material are carried out with the pH adjusted low (e.g., with organic acids) or high (e.g., with NaOH or ammonia) to ensure good solubility (Spiering et al., 2002). Earlier reports had described extraction solvents including mixtures of methanol/ethyl acetate; methylene chloride/ammonium hydroxide, mixtures of organic acids, methanol and methanol/water (see Garner et al., 1993). Rottinghaus et al. (1990) found that a 1:1 methanol:water mixture has the greatest extraction of ergovaline compared to just methanol or water. Because of the other alkaloids often present in tall fescue forage, Spiering et al. (2002) developed a protocol for microanalytical extraction using 2-propanol-lactic acid as extraction solvent to do one extraction for ergovaline and peramine. This protocol was on a micro-scale and allowed analysis of individual plant parts of one plant. However, it was not conducive to large scale extraction and subsequent purification. Presently, routine extraction for analytical purposes use 80% methanol based on the procedure of Yates and Powell (1988). Extraction of tall fescue seed with lactic acid on a 5 kg scale has been accomplished by Moubarak et al. (1993) and yielding a few mg of ergovaline. They concentrated the alkaloid in the lactic acid solution onto Bio-Beads followed by removal with methanol, reducing the methanol and separating the ergovaline on two different HPLC columns. Based on HPLC analysis they obtained egovaline of about 95% purity with very little ergovalinine present. Absence of ergovalinine is significant in testing the activity of ergovaline in bioassays.

Our objective was to isolate and partially purify ergovaline/ergovalinine from up to 150 kg tall fescue seed infected with the endophyte. In the remainder of this report we will use ergovaline to mean both isomers, unless otherwise indicated.

## Materials and methods

Seed contain the greatest accumulation of ergovaline of any tall fescue tissue (Rottinghaus et al., 1991) and we designed several experiments to most efficiently extract the alkaloid. Over time we have used several different batches of seed for these studies, but all had ergovaline levels above 5 mg kg^−1^. Solvent, seed particle size and time used for extraction had significant impact on efficiency of extraction.

We chose 80% aqueous ethanol to do the extractions because it was less expensive to purchase, easier to discard than methanol and the extraction efficiency was similar to methanol. For these extraction studies, seeds were intact or powdered to pass different screen sizes (0.5, 1, 2 mm) and extracted with 80% aqueous ethanol. Materials were extracted for 2, 24, 48, or 150 h. Because of the long extraction time for our large batches the ergovaline/ergovalinine ratio was monitored and decisions were made to minimize the amount of isomerization to ergovalinine.

**Figure 1:**
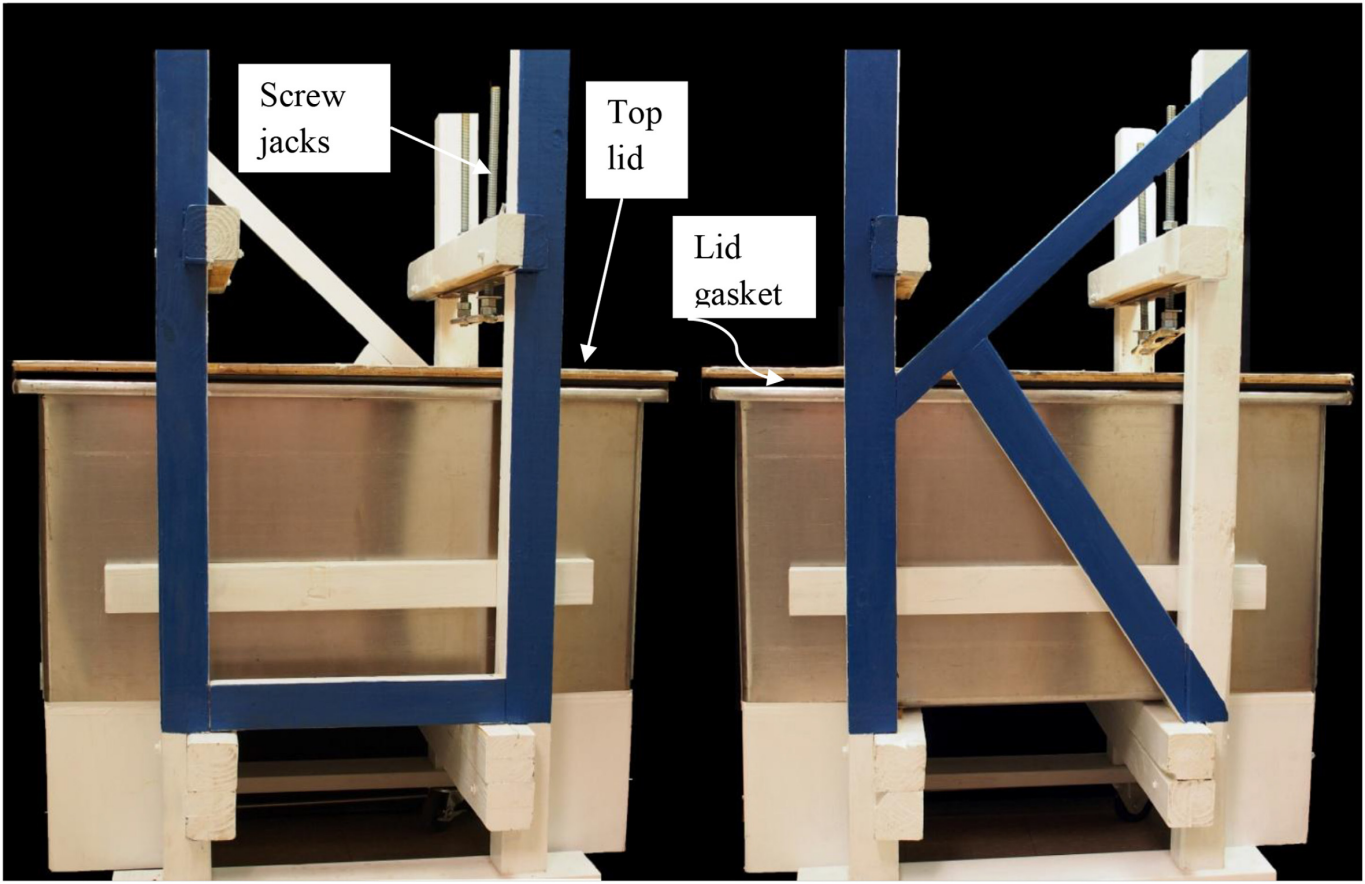
**Front and rear view of tub extractor that holds 150 kg powdered seed**.

For our large scale extractions tall fescue seed that tested high in ergovaline content were ground to pass a 1 mm sieve. Ground seed were packed carefully into a 114 × 52 × 61 cm tub column (Figure 1). There is a gasket around the rim of the tub that seals the top lid to the tub for the introduction of N_2_ to aid in the removal of the extraction solvent. The screw jacks on the top are visible to hold the lid in place while the system is under pressure from the N_2_. The jacks may be used to compress the seed to remove the solvent but we found that using N_2_ was more efficient. On the left end is the exit for the solvent into a tube covered with foil that drains into a large surface area container for the removal of the ethanol from the extract. Packing was done to provide a uniform column substrate as possible. This was done by adding about 25 kg at a time, leveling and lightly packing. Care in packing the column was done to insure that the solvent would migrate uniformly to the bottom of the column. This column holds 150 kg dry powder seed. The bottom of the column was filled with glass marbles above the outlet for solvent. The glass marbles were covered with a metal screen and non-dyed denim filter to keep seed residue from entering the eluate. The seedbed was compressed slightly by 19 mm thick polyethylene plate. Extraction was done with 80% ethanol and to fill the void volume approximately 160 L were added over 18–20 h period. At this time the solvent had reached the bottom of the column and the flow was stopped and seed steeped for 48 h. The tub column was then sealed and compressed N_2_ was introduced onto the top of the tub column to aid in removal of the extracting solvent. The eluate was drained into a large surface area tub and the ethanol removed by a fan blowing on the surface. From the point of emergence from the column and throughout the remaining processing the material was kept in the dark. After elution was complete, approximately 80 L of 80% ethanol was added over an 8 h period. Then the flow was stopped and seed steeped again for 48 h. Elution continued as previously described. After most of the ethanol was removed from the extractor and the drying tub, the resulting extract was freeze-dried to remove remaining ethanol and water and then stored at −20°C.

Purification of the 80% ethanol extract was attempted with many different solvents and combinations. The best protocol in our investigations was one of making a water slurry of the dried 80% ethanol extract followed by partitioning with hexane (discarding the hexane), then partitioning the aqueous fraction with CHCl_3_ (discarding the aqueous fraction) and removal of the CHCl_3_ under vacuum to yield a product with greatly increased ergovaline concentration. All steps were done in brown bottles to limit epimerization.

Purified extracts from the large scale extractions were used in experiments to measure the biological activity in bovine tissues compared to chemically pure ergovaline (Foote et al., 2012, 2013, 2014). Summaries of experimental protocols are provided in the Bioassay section to facilitate explanation of the results.

Routine assay for the alkaloids in each experiment followed a HPLC/FLD (Florescence detector) procedure to quantify ergovaline and ergovalinine concentrations, as developed by Yates and Powell (1988) and modified as in Aiken et al. (2009). Ergovaline and ergovalinine were identified by excitation at 310 and detection at 420 nm with identity confirmed by LC/MS/MS. The *m/z* 223 from parent ion *m/z* 534 was monitored as well the product ion of *m/z* 534 (Lehner et al., 2004).

## Results

Our routine protocol, mentioned above, extracts 500 mg with 10 mL extracting solution while shaking for 2 h. Whole seed were extracted for 48 h with shaking and the powdered seed were extracted via our routine procedure. The smaller the particle size the greater amount of ergovaline that was extracted and the ergovaline/ergovalinine ratio did not change significantly (Table 1). Ergovaline/ergovalinine ratio ranged from 64/36 to 61/39. Even extracting whole seed for 48 h only removed about 5% of the alkaloid. The assumption that all the ergovaline was extracted in the first step of the ground seed is not valid but is about 84% efficient for the finest particle size and exhaustive extraction. Because grinding large amounts of tall fescue seed through a 0.5 mm screen is not reasonable (time-wise) for large-scale extractions (kg amounts), we used the 1 mm particle size for our large-scale extractions.

**Table 1:** **Seed particle size and extraction efficiency with 80% aqueous ethanol**.

**Sample identification**	**Ergovaline**		**Ergovalinine**		**Total extracted**
	**μg g^−1^**	**stdev**	**μg g^−1^**	**stdev**	**(%)**
Whole seed^1^	0.30	0.04	0.47	0.06	5
**GROUND THROUGH**					
2 mm screen	2.24	0.12	1.42	0.08	34
1 mm screen	3.79	0.14	2.26	0.08	60
0.5 mm screen	5.19	0.13	3.32	0.20	84

1 *Whole seed were extracted for 48 h, other sizes were extracted for 2 h*.

The interaction between particle size and time of extraction demonstrated that increased shaking extraction time, regardless of the particle size increased ergovaline extraction (Table 2). Even at the 0.5 mm particle size the increased time from 2 to 48 h increased ergovaline extracted into the ethanol. The increased efficiency of extraction for the 2, 1, and 0.5 mm particle size from 2 to 150 h was 251, 61, and 19%, respectively.

**Table 2:** **Extraction time, particle size interaction for efficient extraction with 80% ethanol**.

**Particle size**	**Extraction time**	**Ergovaline**		**Ergovalinine**	
**mm**	**h**	**μg g^−1^**	**stdev**	**μg g^−1^**	**stdev**
2	2	2.24	0.12	1.42	0.08
2	24	4.27	0.06	2.83	0.13
2	48	5.00	0.37	3.26	0.21
2	150	5.30	0.16	3.36	0.07
1	2	3.79	0.14	2.26	0.08
1	24	4.92	0.16	3.12	0.15
1	48	5.50	0.01	3.49	0.04
1	150	5.94	0.07	3.82	0.08
0.5	2	5.19	0.13	3.32	0.20
0.5	24	5.80	0.05	3.78	0.14
0.5	48	6.10	0.07	3.96	0.01
0.5	150	6.19	0.02	3.91	0.12

**Figure 2:**
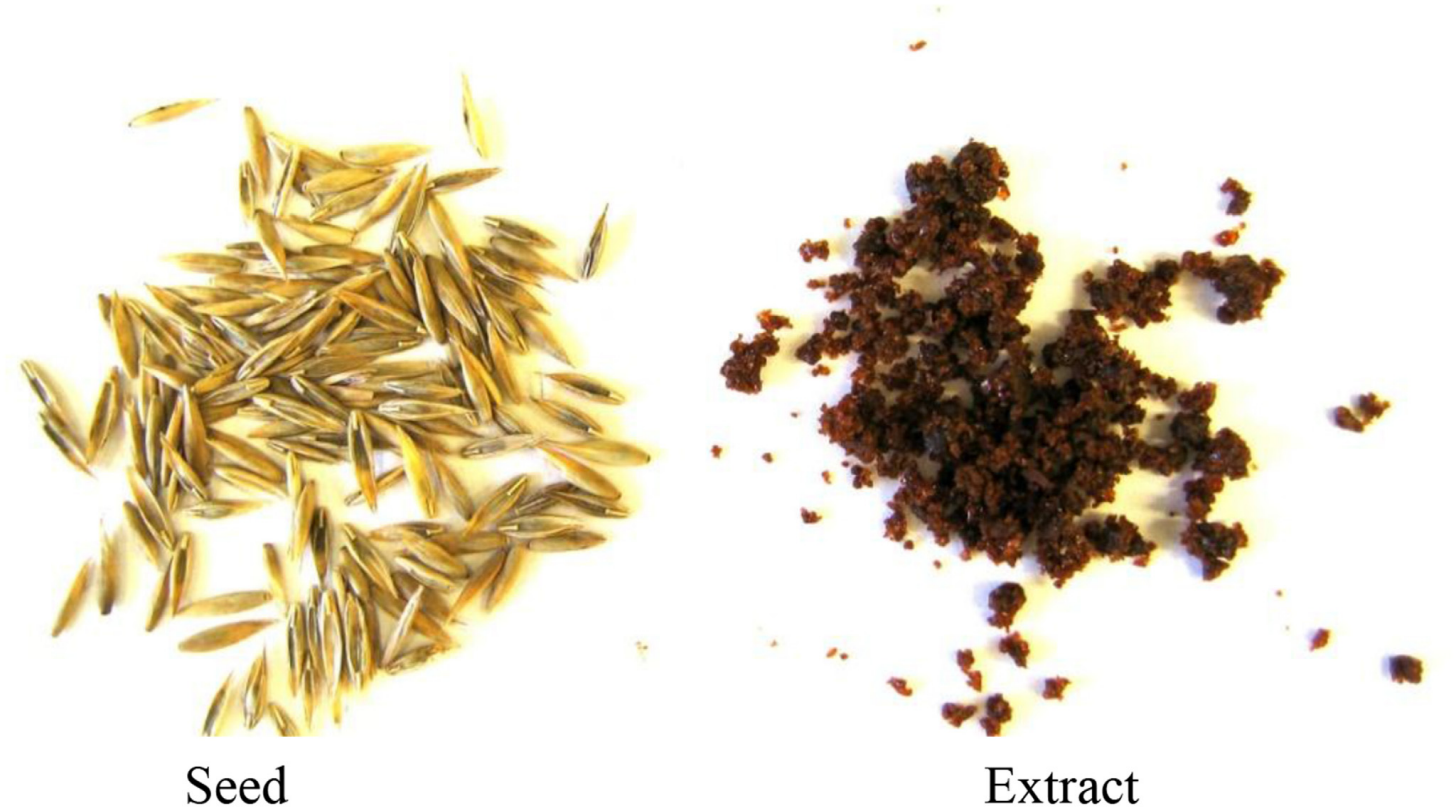
**Seed and initial dried extract**.

From 150 kg of seed, we obtained 8.3 kg of dried extract. This extraction resulted in an 80% recovery of ergovaline and an 18-fold increase in concentration. Initial seed and the resulting freeze-dried extract are depicted in Figure 2. Because we had to use the longer times of extraction for our large batch extractions, we measured the epimerization that may occur between ergovaline and ergovalinine in the solvents to be used. Chemically pure ergovaline dissolved in 80% methanol had no measureable epimerization immediately after being dissolved. However, by 22 and 47 h significant epimerization occurred, 16 and 26 %, respectively (data not shown). Similarly, seed extract solubilized in 80% ethanol (Table 2) or acetonitrile (data not shown) also had significant epimerization, about 40% conversion and was very consistent across treatments during extraction and measurement.

To obtain the best efficiency of recovering ergovaline from the large scale dried extract we tried several solvents, solvent sequences and solvent combinations to get efficient recovery and further purification. Any approaches that utilized water resulted in high recovery of lysergic acid and isolysergic acid but little ergovaline recovery. A water extract followed by CHCl_3_ removed much of the ergovaline and two CHCl_3_ extracts removed most of the ergovaline (the first 4 rows) were done in this sequence on one sample and results summarized (Table 3). Chloroform without some water wetting of the dried extract removed much smaller portion of the ergovaline. Acidic and basic solutions with organic solvents plus hexane and methyl-tert butyl ether were also tried for extraction of ergovaline from the initial dried extract, but were less effective than CHCl_3_. However, initially extracting with hexane from a water slurry of the freeze dried extract to remove lipophilic substances greatly increased the purification of the CHCl_3_ extract from the slurry following the hexane extraction. The hexane fraction did contain 10–15% of the ergovaline (bottom Table 3) and this could be back extracted with 80% methanol to improve overall efficiency, if needed. The CHCl_3_ fraction was filtered and CHCl_3_ removed under vacuum at 25°C in the dark. Dried residue was solubilized in 80% aqueous methanol.

**Table 3:** **Purification attempts from initial seed extract**.

**Extraction solvent**	**Lysergic acid**	**Isolysergic acid**	**Ergovaline**		**Ergovalinine**		**Lysergic acid/isolysergic acid**	**EV/EVI**
	**Recovery**			**stdev**	**Recovery**	**stdev**		
	**%**	**%**	**%**		**%**		**Epimer portion**	**Epimer portion**
1st water	62	51	9		12		56/44	63/37
2nd water	5	4	1		1		55/45	56/44
1st CHCl_3_	1	5	63	5	67	8	9/91	64/36
2nd CHCl_3_	0	2	16	2	15	1	0/100	64/36
only CHCl_3_	ND	ND	37		45			58/42
**H_2_O/HEXANE/CHCl_3_**								
Hexane fraction	6	6	15	1	12	2	51/49	71/29
CHCl_3_ fraction	ND	ND	68	6	65	3		61/39
80% methanol	100	100	100		100		51/49	62/38

*Measures of lysergic acid and ergovaline recoveries at different steps of the purification. ND, not detected*.

**Figure 3:**
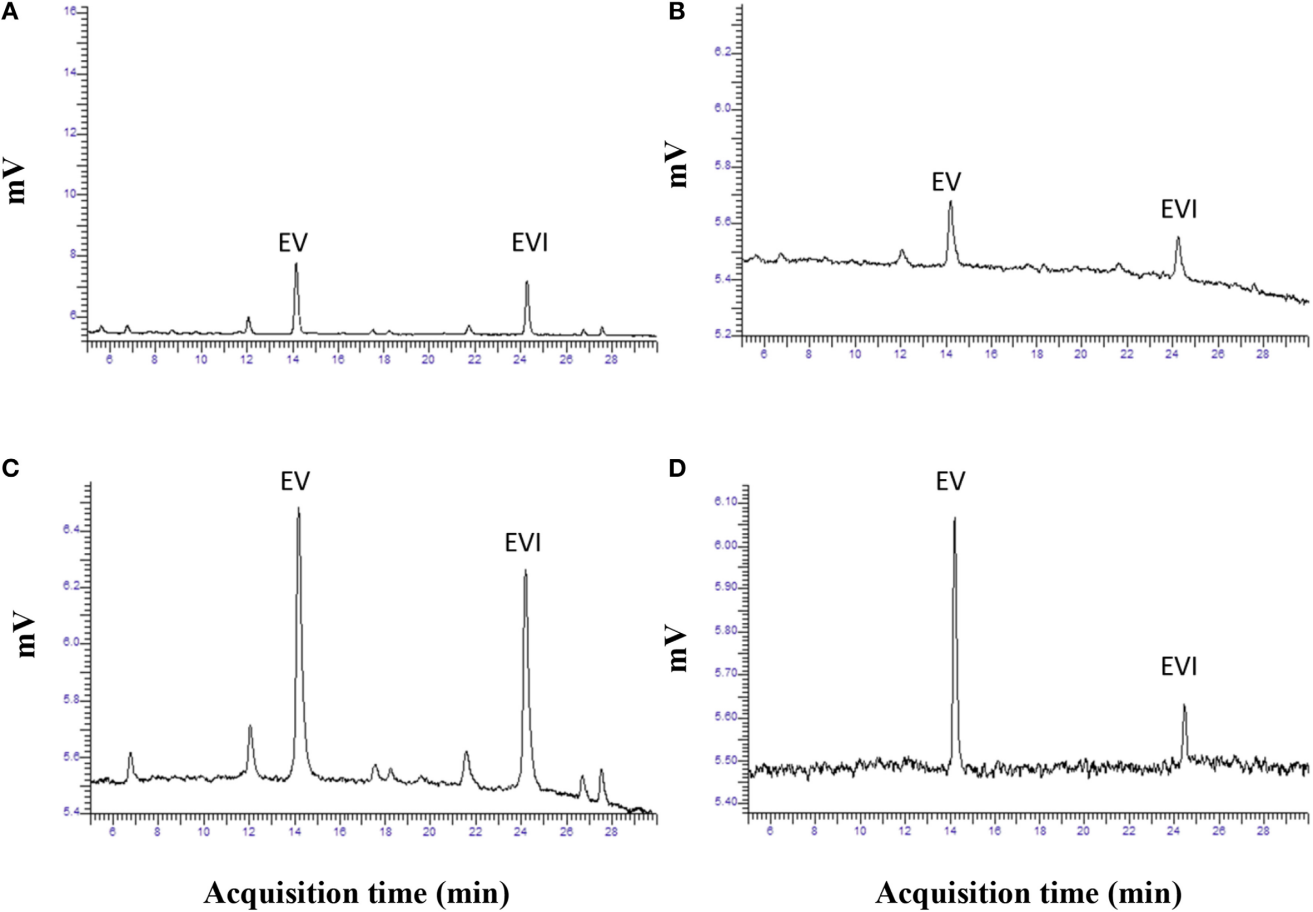
**Chromatograms of ergovaline from initial seed to a partially purified material**. Panel **(A)** is chromatogram of routine analysis of powdered seed (EV, ergovaline; EVI, ergovalinine). Panel **(B)** is a chromatogram from dried material following 80% ethanol extraction. Panel **(C)** is chromatogram of dried material following the water/hexane/CHCl_3_ purification. Panel **(D)** is a chromatogram of HPLC purification of ergovaline from **(C)**.

**Figure 4:**
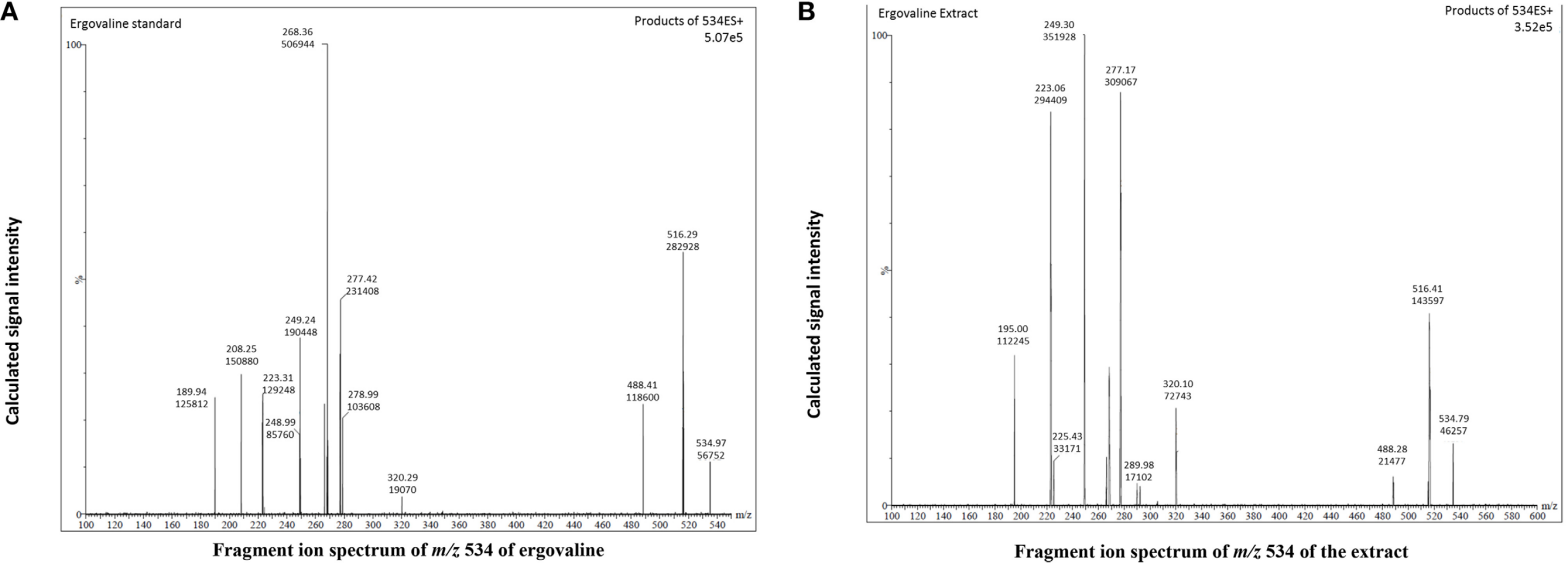
**Fragment ion spectrum of *m/z* 534 fragment of chemical standard ergovaline and the partially purified extract from panel 3D**. **(A)** is chemical standard for ergovaline, **(B)** is the ergovaline extract.

Based on the above results we selected the following protocol for the second step in purification. Three hundred mL of water was added to 600 g of the dried extract and mixed to a smooth slurry to remove all “crystalline” pieces. Slurry was added to a 4 L brown bottle and 1800 mL of hexane was added. The mixture was vigorously shaken for 10 min and the hexane and aqueous layers were allowed to separate. This could take up to 2 h. Hexane removed many of the lipids and the alkaloids remained in the aqueous fraction. The hexane layer was decanted and the aqueous fraction was extracted with hexane two more times. The aqueous fraction was extracted with 1800 mL chloroform 3-times and the chloroform fractions combined and removed in a rotary evaporator. Residue was stored at −20° C for use. Ergovaline concentration increased from the seed about 350- to 400-fold (Figures 3A–C). Chromatogram in Figure 3A is from initial seed extract with ergovaline (EV) and ergovalinine (EVI). The initial crude 80% ethanol extract alkaloid content is illustrated in Figure 3B. This chromatogram is a 100-fold dilution of the extract for comparison with Figure 3A. Ergovaline present in the CHCl_3_ of the water/hexane/CHCl_3_ cleanup had no lysergic acids and the solution for the chromatogram had been diluted 600-fold (Figure 3C, Table 3). Lysergic acid and loline alkaloids were not detected in the final purified extract (not shown on the chromatograms). Fragment ion spectrum of ergovaline and of the partially purified ergovaline *m/z* 534 fragment are indicative of ergovaline (Figure 4). Further purification may be done with HPLC separation and collection of ergovaline and ergovalinine. The HPLC purification of the water/hexane/CHCl_3_ extract is illustrated in Figure 3D. The ergovalinine is most likely from epimerization that occurred during the chromatography or processing. A final purification by HPLC was reported earlier by Moubarak et al. (1993).

## Bioassay of purified extract

**Figure 5:**
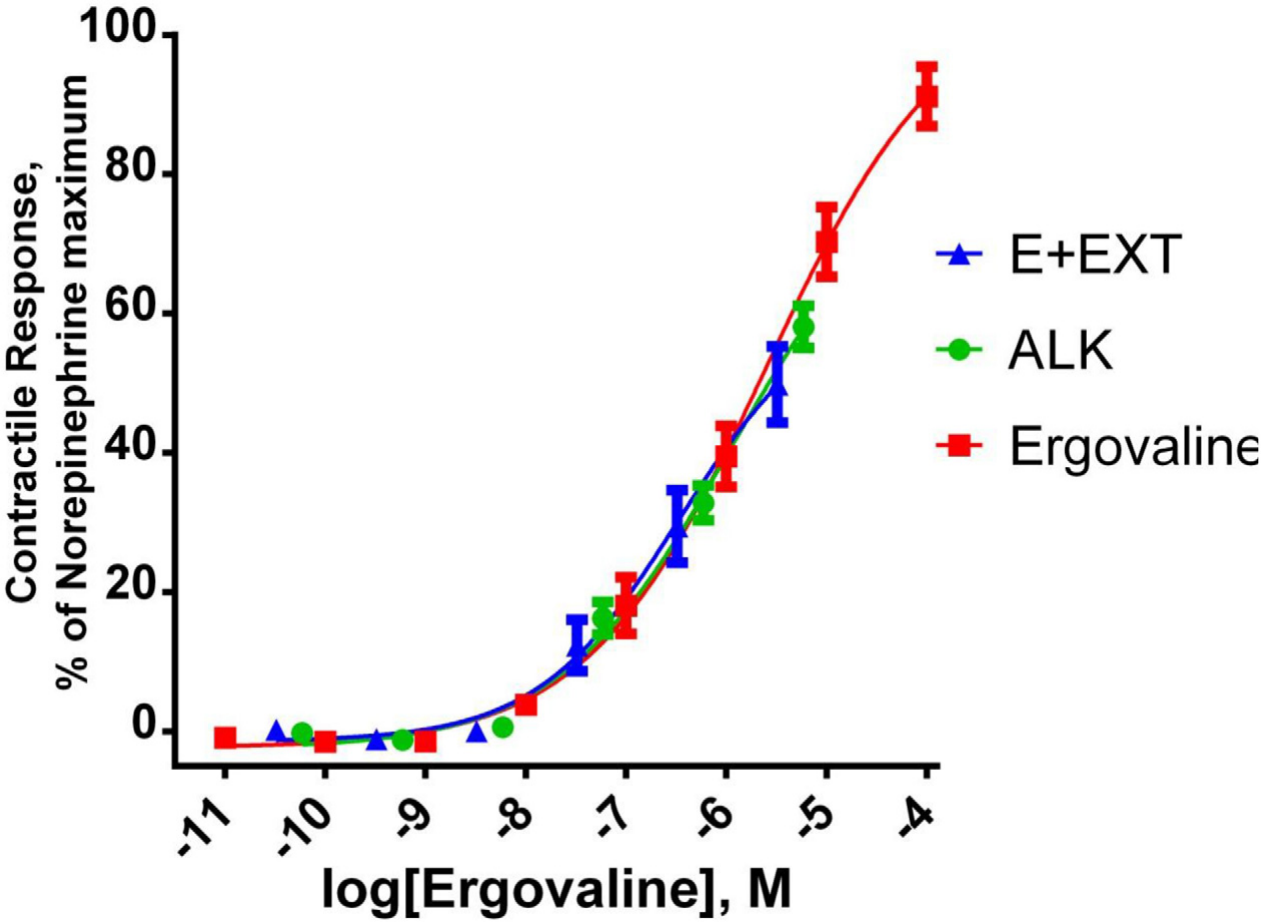
**Contractile response of bovine lateral saphenous vein**. Response of bovine lateral saphenous vein to ergovaline, an endophyte-infected tall fescue seed extract (E+EXT) standardized to ergovaline concentration, and a mixture of ergot alkaloids (ALK) that reflects the alkaloid profile of E+EXT. Regression lines represent the fitting of data to a sigmoidal concentration response curve. The ALK treatment was prepared by dissolving ergovaline, ergotamine, α-ergocryptine, ergocristine D-lysergic acid hydrate, and ergonovine in 80% methanol. The alkaloid concentrations of this ALK treatment were based on alkaloids quantified in the E+EXT. E+EXT was prepared as described above for purification of the crude extract from the tub extractor. All treatment solutions were based on the ergovaline concentration. The other alkaloids in the E+EXT and the ALK mimic solution were in the 10^−8^ to 10^−10^ M. Figure taken from Foote et al. (2012).

Purified extracts were used in experiments to measure the biological activity in bovine vasculature. An experiment was conducted to determine if substances extracted from endophyte infected tall fescue other than ergovaline were responsible for vasculature responses *in vitro* (Foote et al., 2012). Lateral saphenous veins from cattle were tested in a multi-myograph with different alkaloid treatments. Alkaloid treatments were (1) chemically pure ergovaline (EV), (2) endophyte infected seed extract (E+EXT); (3) a mixture of alkaloids (ALK) that mimicked those found in the E+EXT, and (4) an extract from endophyte free seed extract. An extract was generated using endophyte containing seed described above and a second similar extract was generated from endophyte-free seed. The partially purified residue from each extract was solublized in 50 mL of 80% methanol. The endophyte-free extract was diluted in the same manner as for the extract from the endophyte-infected seed. The amount of each extract and chemical alkaloid treatment added to myograph cells were determined by amount of ergovaline required meet the ergovaline concentration along the X-axis (Figure 5). Ergovaline/ergovalinine ratio of the alkaloid extract was 60/40. A mixed alkaloid treatment was prepared by dissolving ergovaline, ergotamine, α-ergocryptine, ergocristine D-lysergic acid hydrate, and ergonovine in 80% methanol. The alkaloid concentrations of this mixed alkaloid treatment were based on alkaloids quantified in the endophyte infected seed extract, including ergovaline. Both the E+EXT and ALK mixture induced similar contractile response as ergovaline. Lack of a significant response from the endophyte-free seed extract (data not shown) and the fact that the response from the E+EXT and ALK were similar to the chemically pure ergovaline response (Figure 5) suggests that ergovaline is the only substance in the endophyte-infected tall fescue extract that is causing the contractile response in this bioassay, and perhaps in any of the affected vasculature. The seed extract also decreased *in vivo* reticuloruminal epithelial blood flow about 50% and volatile fatty acid absorption (acetate, propionate and butyrate) from the washed reticulorumen (Foote et al., 2013). Acute exposure of *in vitro* bovine rumen epithelium to the extract had no effect on acetate or butyrate flux (μmol/cm^2^ h) across the epithelium (Foote et al., 2014).

## Discussion

Data in Tables 2, 3 indicate that chemically pure ergovaline does not epimerize as rapidly as seed extracts that contain many other substances in addition to ergovaline in our studies. Our data are not exactly comparable as the pure ergovaline that was is in 80% methanol and the seed extracts are in 80% ethanol. Smith and Shappell (2002) reported that in methanol, ergovaline had about 20 and 45% epimerization in 22 and 47 h, respectively. In water, they reported less, about 15 and 30%, epimerization over the same time periods. Both their studies were done at 37°C which would enhance the rate of epimerization. The water and lower temperature (~23°C) in our study probably resulted in the lesser rate of epimerization measured. We did not measure the rate of epimerization of ergovaline in 80% aqueous ethanol but it was stable at about 60/40 ergovaline/ergovalinine over the time periods of our studies (Tables 2, 3). Product ion fragments of *m/z* 534 of the partially purified extract (Figure 4B) are indicative of ergovaline and agree with previous mass spectrum published (Lehner et al., 2004) and the current product of purification is biologically active. In different bioassays the partially purified extract resulted in similar biological activity based on the amount of ergovaline in the extract compared to chemical ergovaline.

## Conclusions

Solvent, seed particle size, and time used for extraction had significant impact on efficiency of extraction. Overall this is a protocol for extraction of large amount of high ergovaline plant tissue that yields dried extracts with enhanced levels of ergovaline (350- to 400-fold increase) that are biologically active. Activity is equal to ergovaline alone in selected bioassays. Additional purification was achieved with HPLC separation of ergovaline and ergovaline. This further purification of ergovaline will be useful in specific cellular bioassays and for analytical purposes.

## Author contributions

All authors were involved in aspects of data acquisition, analysis, and interpretation. All authors contributed to writing the manuscript, approved the final version, and are accountable for the data presented and interpretation therein.

### Conflict of interest statement

The authors declare that the research was conducted in the absence of any commercial or financial relationships that could be construed as a potential conflict of interest.
